# Theta oscillations mediate pre-activation of highly expected word initial phonemes

**DOI:** 10.1038/s41598-018-27898-w

**Published:** 2018-06-22

**Authors:** Irene F. Monsalve, Mathieu Bourguignon, Nicola Molinaro

**Affiliations:** 1 BCBL, Basque center on Cognition, Brain and Language, Donostia/San Sebastian, San Sebastian, Spain; 2 UPV/EHU, Universidad del Pais Vasco, San Sebastian, Spain; 3 Laboratoire de Cartographie fonctionelle du Cerveau, UNI – ULB Neuroscience Institute, Université libre de Bruxelles (ULB), Brussels, Belgium; 4 Laboratoire Cognition Langage et Développement, UNI – ULB Neuroscience Institute, Université libre de Bruxelles (ULB), Brussels, Belgium; 5 Ikerbasque, Basque Foundation for Science, Bilbao, Spain

## Abstract

Prediction has been proposed to be a fundamental neurocognitive mechanism. However, its role in language comprehension is currently under debate. In this magnetoencephalography study we aimed to find evidence of word-form phonological pre-activation and to characterize the oscillatory mechanisms supporting this. Participants were presented firstly with a picture of an object, and then, after a delay (fixed or variable), they heard the corresponding word. Target words could contain a phoneme substitution, and participants’ task was to detect mispronunciations. Word-initial phonemes were either fricatives or plosives, generating two experimental conditions (expect-fricative and expect-plosive). In the pre-word interval, significant differences (α = 0.05) emerged between conditions both for fixed and variable delays. Source reconstruction of this effect showed a brain-wide network involving several frequency bands, including bilateral superior temporal areas commonly associated with phonological processing, in a theta range. These results show that phonological representations supported by the theta band may be active before word onset, even under temporal uncertainty. However, in the evoked response just prior to the word, differences between conditions were apparent under variable- but not fixed-delays. This suggests that additional top-down mechanisms sensitive to phonological form may be recruited when there is uncertainty in the signal.

## Introduction

Context and prior knowledge strongly influence perception. Language processing is no exception to this, with decades of neuro- and psycho-linguistic research showing contextual facilitation of comprehension, both in behavioural and neural measures. Words that are predictable within a given sentential context are read faster or skipped more often^1,2^, and elicit a reduced neural response as measured by the N400 event related potential (ERP) component^3^. Furthermore, ERP modulations by predictability have also been found in earlier components^4–7^ (100–200 ms) associated with perceptual processing.

However, the nature of the cognitive mechanisms supporting contextual facilitation of speech comprehension, and the representational level at which they may operate are currently under heated debate^8,9^. One of the contended aspects is whether semantic-level contextual constraints may facilitate phonological (or orthographic) processing of predicted words through word-form pre-activation (before bottom-up information is available), or through faster selection of possible candidates once bottom-up input has arrived. Answering this question has been hindered by the fact that most studies of prediction in language have focused on post-target word interval, rendering both interpretations plausible.

Two types of paradigms present exceptions to this general trend. The first are ERP studies focusing on the article preceding a highly expected noun whose form (e.g. ‘a’ vs. ‘an’) depends on the phonological features of the upcoming noun^10,11^. In these studies, the N400 response to the article was found to be larger when it did not agree with the expected noun, showing that the phonological representation of the latter must have been pre-activated. Although these effects show that phonological pre-activation is indeed possible, their robustness and generalizability have been brought into question^12^. Furthermore, the neural evidence they provide still reflects the effects of an unmet prediction, rather than the prediction generation process itself.

The second type of studies have actually looked at the pre-target word interval by analyzing the preparatory activity before a contextually highly expected printed word compared to a less predictable counterpart. In a picture-word matching paradigm, Dikker and Pylkkanen^13^ found increased magnetoencephalography (MEG) activity in the theta band (4–7 Hz) before word onset in the left mid-temporal and, successively, in visual cortex, when the previous picture represented a single object as compared to a picture of a group of objects. This finding was interpreted as evidence of lexical retrieval (given the role of the mid-temporal gyrus in lexical access^14,15^) and visual feature pre-activation, when the identity of the upcoming word could be predicted. Molinaro and colleagues^7^ compared EEG activity before target words embedded in multi-word fixed expressions, or in low-constraining sentences, finding increased functional connectivity in the theta band between frontal and occipital electrodes for the former, suggesting top-down modulation of sensory cortices prior to word onset when the target could unequivocally be anticipated. Fruchter *et al*.^16^, in a MEG study, used adjective-noun pairs where the adjective provided the context for the following noun. They focused their analysis on a middle temporal gyrus area, finding an increased response in evoked activity to highly predictive adjectives and a subsequent decreased response to the highly predicted noun. They interpret this finding as a lexical pre-activation of the expected noun, but whether this led to an orthographic (visual) pre-activation was not examined. Cope and colleagues^17^, (see also^18^). provide compelling evidence of predictive top-down frontal contributions to speech processing mediated through neural oscillations in the beta band. Using auditory words with different levels of sound degradation and prior written words as cues with 50% validity, they show a delayed reconciliation between prior predictions and sensory input in the patients with frontal lobe degeneration as compared to the controls. Importantly, they show that pre-stimulus beta band activity (~24 Hz) relates to the behavioral evaluation of the match between prime and target.

By looking at the pre-target word interval, these studies examined the neural substrates of the prediction process itself and have provided suggestive evidence of word-form pre-activation. However, they all share one important limitation. The enhanced activity in early sensory cortices found before a highly expected word as compared to a less expected one could be explained in terms of different allocation of processing resources when semantic information can guide subsequent visual analysis, without involving word-form pre-activation per-se. Furthermore, to the best of our knowledge, evidence for pre-target word activation is still scarce, and has come only from studies where words are presented visually. Although top-down influences on sensory areas have been well documented in the auditory domain^18,19^, these have always focused on response to the actual word and have thus examined processing differences once initial sensory input is available.

**Figure 1:**
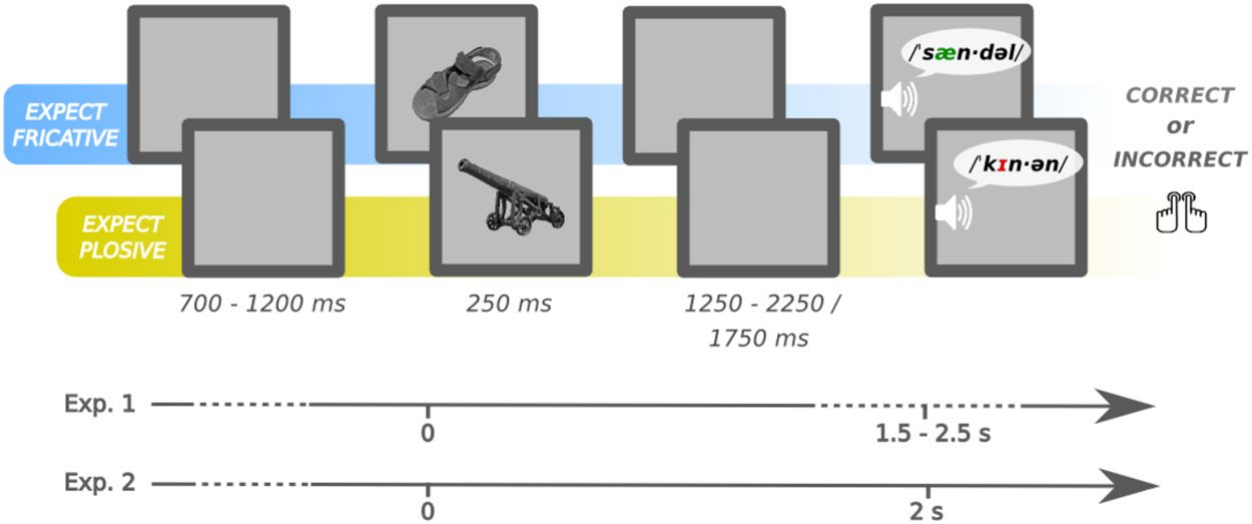
Experimental paradigm. Object pictures served as predictive cues to an auditory word presented after a delay. The word, always congruent with the picture, could contain a mispronunciation which participants had to detect. The phonological properties of the word’s initial phoneme were manipulated to create two experimental conditions: expect-fricative (/f/, /s/, /θ/) and expect-plosive (/k/, /p/, /t/). Participants were not made aware of this manipulation. This paradigm was implemented in two separate experiments, with either fixed or variable image-to-word delays. English examples are provided in the figure for clarity, but actual stimuli were in Spanish (see Table 3 for full stimuli lists). Object pictures in the figure, as those making up the experimental stimuli, were extracted from the BOSS database [46, CC-BY-SA license: https://creativecommons.org/licenses/by-sa/3.0/].

The main aim of the present study is to contribute to the body of research examining the pre-target word interval by focusing on the auditory domain using MEG, and to go beyond predictive vs. non-predictive contrasts by comparing activity between two highly expected words that differ in their word-form (phonological) features: words starting either with a fricative or a plosive. If prior expectations involve pre-activation of word-form representations, we should be able to find differences between them before stimulus onset, in cortical areas involved in phonological processing (superior temporal gyrus/sulcus, STG/STS^15,20^). Pre-activation may also depend on temporal predictability (*when* the target will appear) about upcoming words. Given the importance of temporal information for the oscillatory mechanisms supporting speech comprehension, temporal predictability might be required to observe phonological-feature pre-activation. On the other hand, it has been suggested that uncertainty regarding the bottom-up input could enhance reliance on top-down predictive mechanisms^9^. Therefore, we also manipulated the temporal predictability of word onset by conducting two versions of the same experiment. Hence, the target words were always highly expected but their onset was either variable (experiment 1) or fixed (experiment 2) with respect to a visual cue that set up the expectation (see Fig. 1).

We set out to characterize the neural signature of the predictive process, by assessing the activity preceding expected words. We first focused on evoked activity around word presentation to evaluate 1) whether the phonological contrast employed elicited statistically significant differences (differential response ~100 ms post-stimulus onset) and 2) whether baseline state is perturbed by the prediction process^13^. We then evaluated how ongoing oscillations following image presentation reflect the building up of the prediction process. Indeed, neuronal oscillations have been implicated in different top-down mechanisms^21^, such as attentional selection through alpha inhibition^22,23^ and prediction regarding timing and stimulus identity through the beta band^24^. Furthermore, cortical oscillations have been shown to play a key role in speech comprehension through entrainment to input stream regularities^25^. If predictive processes involve the generation of word-form representations before stimulation, we expect to find differences between fricative and plosive conditions in the interval preceding the target word. We expect these to manifest in low-frequency oscillations (<30 Hz), and to arise in superior temporal cortices.

## Results

### Behavioral data

Participants in both experiments had to detect mispronunciation of the target words and showed high accuracy (mean of 95% in both experiments), varying between 85 and 100% in experiment 1, and 80 and 99% in experiment 2. Mean response time across experiments and conditions was 750.3 ms (with a standard deviation of 67.5 ms).

We attempted to fit maximal mixed-effects models, using reaction times as the dependent variable and our main experimental manipulations and their bivariate interactions as fixed effects and as by-subject random slopes, in addition to random by-subject and by-item intercepts. This maximal model did not converge. We subsequently removed the by-random slopes for the interaction terms in an intermediate model and built a final model in which only interactions with significant fixed effects (|t-value| > 2) were added as random slopes (see Table 1 for final model specification).

**Table 1:** Fixed effects for RT model.

Fixed effect	Estimate (ms)	Standard error (ms)	t-value
**Intercept**	**722.0**	**18.6**	**38.9**
**Phoneme (plosive)**	**−53.5**	**16.2**	**−3.3**
Experiment (2)	−3.8	22.7	−0.2
**Cue (non-predictive)**	**141.9**	**19.9**	**7.5**
**Pronunciation (wrong)**	**15.8**	**9.3**	**1.7**
Phoneme (plosive):Experiment (2)	−3.7	5.2	−0.7
Phoneme (plosive):Cue (non-predictive)	1.9	22.5	0.1
Phoneme (plosive):Pronunciation (wrong)	4.5	4.9	0.9
Experiment (2):Cue (predictive)	−3.1	13.5	−0.2
Experiment (2):Pronunciation (wrong)	5.0	11.3	0.4
**Cue (non-predictive):Pronunciation (wrong)**	**19.4**	**6.5**	**3.0**

Highlighted rows indicate significant (|t| > 2) and marginally significant predictors in final model. The predictors, all bivariate categorical variables were coded using treatment coding, making plosive, predictive, correct, Experiment 1 trials as the reference level. Final model specification: RT ~ Phoneme *(Experiment + Cue + Pronunciation) + Experiment* (Cue + Pronunciation) + Cue * Pronunciation + (1 + Phoneme + Experiment + Cue + Pronunciation + Cue * Pronunciation | subject) + (1 | Cue image).

This final model showed clear modulations of reaction times by the predictive value of the image cue and by initial phoneme condition, being slower for non-predictive than predictive image-cues and for fricative- than plosive-initial words (see Table 1). There was a marginal effect of pronunciation, with incorrect words receiving slower responses than their correct counterparts, and an interaction between predictiveness and pronunciation, with the mispronunciation effect being considerably larger for trials with non-predictive than predictive image cues. Temporal predictability of word onset did not influence reaction times, with no significant effect of experiment or its interactions with the other independent variables.

### Neural response to expected target words: word-locked event-related fields

Before examining the pre-target word oscillatory activity, we examined the event-related fields (ERFs) around word onset. Both pre- and post- word intervals were included to specifically look for evidence of prediction-related baseline perturbation and for difference in auditory responses to words.

We performed statistical inference at the sensor-level using cluster-based permutations over a 500 ms interval centered around word onset. We assessed the contrast between expect-fricative and expect-plosive conditions, using the 41 subjects of both experiments pooled together. This contrast will be later referred to as the phoneme expectation contrast, or the phoneme expectation effect when deemed significant. We then evaluated whether phoneme expectation effects are modulated by the degree of temporal predictability (i.e., whether there is a temporal predictability effect), by comparing the phoneme expectation contrast between the two experiments. Finally, we performed source reconstruction with minimum norm estimate (see Methods section) to identify the brain regions underpinning the effects identified in sensor data.

**Figure 2:**
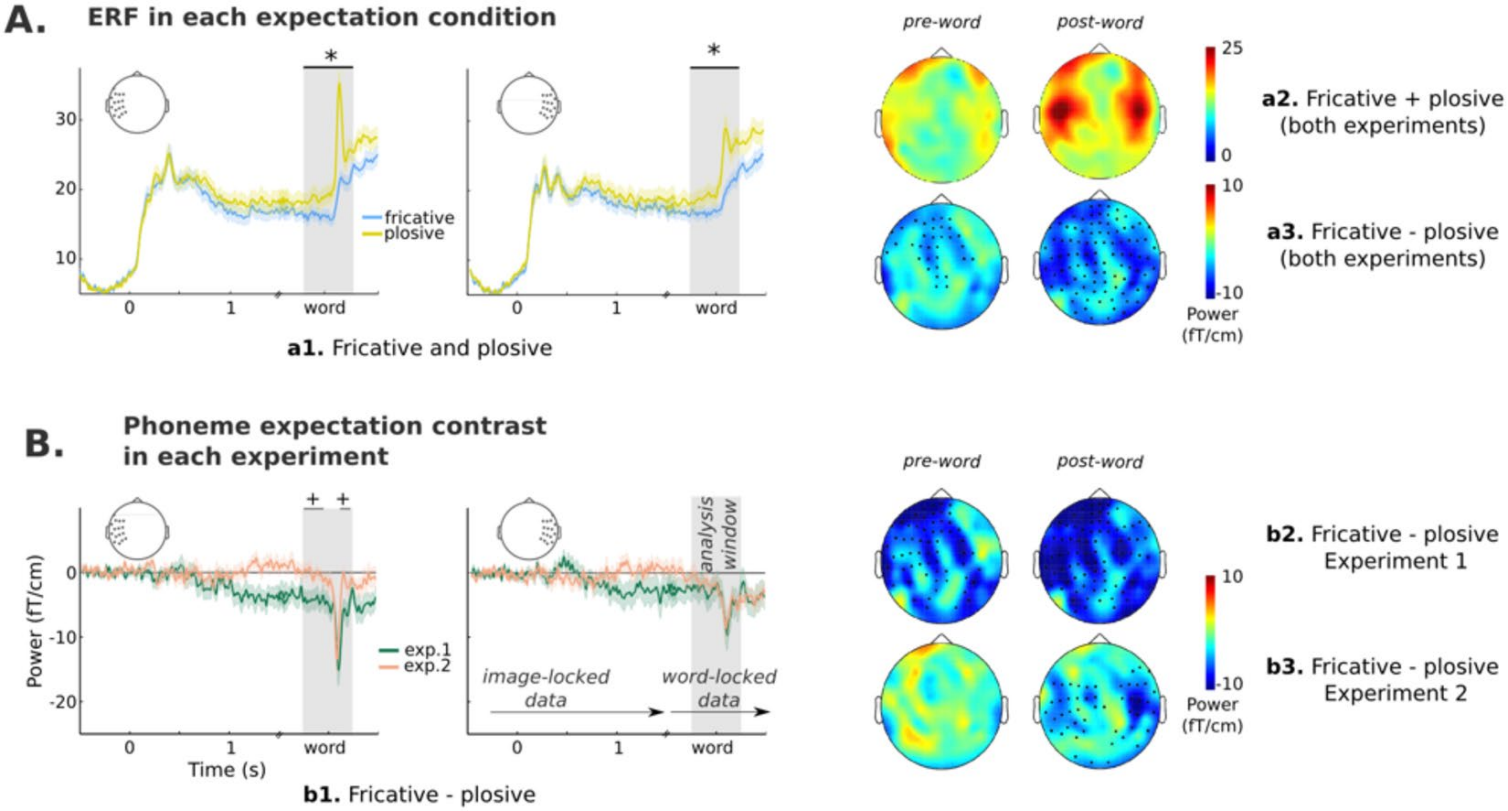
Peri-word ERF response. (**A**) Phoneme effect with subjects of both experiments pooled together (N = 41). On the left, the time-course plots show the averaged evoked response over left and right temporal sensors for each phoneme condition. The depicted sensors were chosen for illustrative purposes only, on the basis of an a-priori parcellation of the sensor space. Shaded area indicates the temporal window used in the statistical analysis, and black horizontal line within this area shows the temporal extent of the clusters reported in the results section (‘+’ indicates a *p*−*value* < 0.1, and ‘*’ < 0.05). On the right plots *a*2 and *a*3 indicate the topography of the evoked response during the pre- and post-word segments of the analyzed window. (**B**) Interaction between phoneme and temporal uncertainty. On the left, plot *b*1 shows the time-course of the difference between phoneme conditions in each experiment (N = 20, 21) over left- and right-temporal sensors. On the right, plots show the topography of the phoneme expectation effect (i.e., the contrast between ERFs to fricative and plosive conditions) separately for each experiment (N = 20, 21). Sensors that formed part of the clusters with *p* < 0.1 are marked in black. The time-course plots include a shaded area around the mean indicating the standard error (for visualization purposes). The time-courses (*a*1 and *b*1) were constructed using image-locked data from the beginning of the interval up to 1600 ms, and word-locked data from 500 ms prior to word onset. 100 ms of overlap are thus present and marked as a discontinuity in the x-axis.

Word presentation elicited a marked amplitude increase over mid bilateral sensor regions, that was more pronounced for plosive- than fricative-initial words (see Fig. 2A). Cluster-based permutations comparing the brain response to fricatives and plosives (all 41 subjects) confirmed that these differences were significant (largest cluster *p* = 0.003, second largest cluster *p* = 0.2). Interestingly, this phoneme expectation effect seemed to start before word onset (the largest cluster spanned the whole analysis time window).

The test for an interaction between phoneme expectation and temporal predictability revealed a marginally significant effect (largest cluster −250 to −160 ms, *p* = 0.07; second largest cluster *p* = 0.09) that started before word onset and was evident bilaterally over temporal and frontal regions (see Fig. 2B). This marginal interaction seemed to be driven by a pre-target word phoneme expectation effect that was apparent only when word onset was variable (i.e., in experiment 1). Furthermore, this effect is present even before our analysis time window (see Fig. 2B) and seems to persist after word onset.

**Figure 3:**
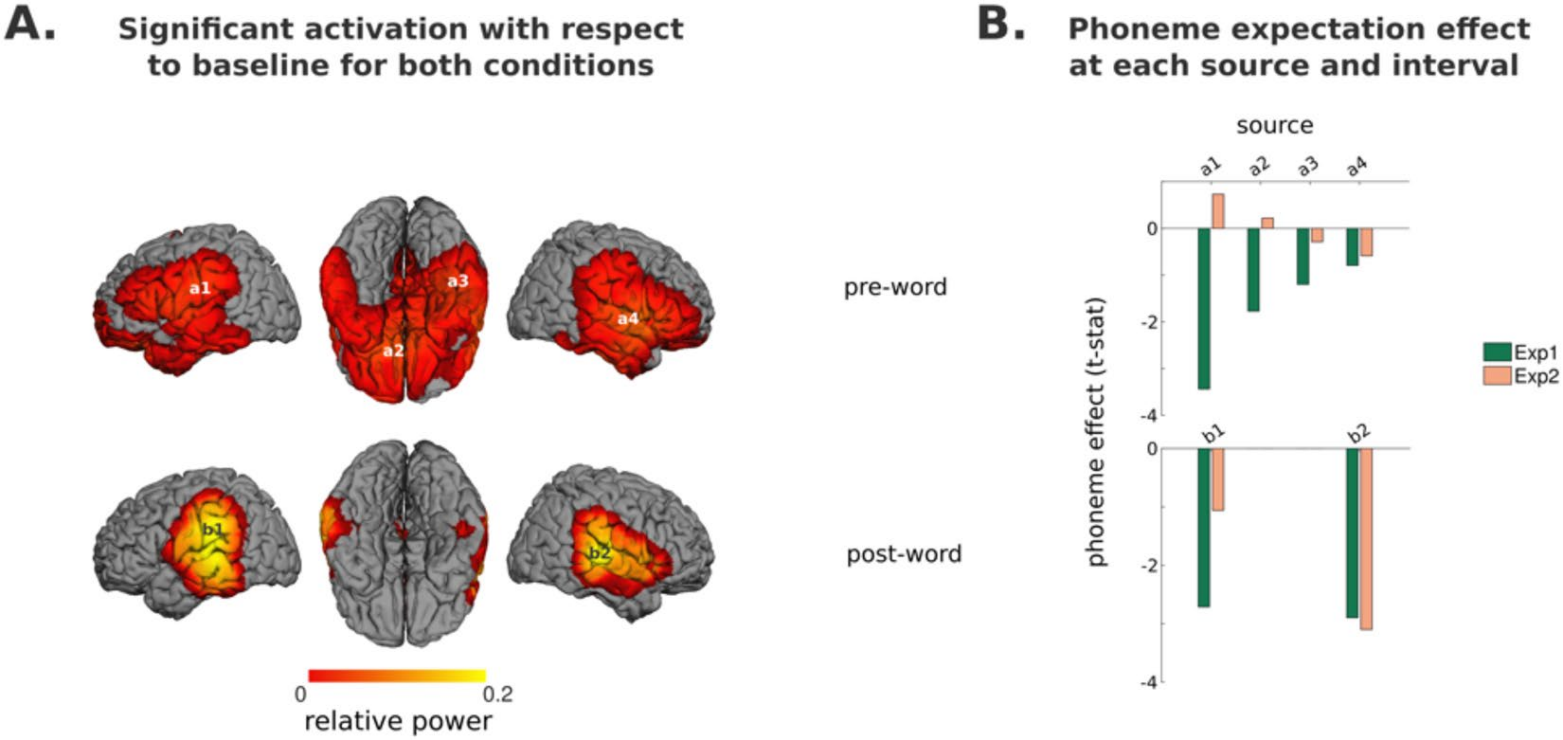
Source reconstruction of ERF effects. (**A**) Regions of significant power change with respect to baseline (both conditions and experiments, N = 41) at 250 ms windows just before and after word onset. The peak activity locations within these areas are marked (*a*1 to *b*2) within these images. (**B**) Effect-size of the phoneme-expectation effect (estimated as the standardized difference between conditions) at each peak and time interval for each experiment separately (N = 20, 21).

We performed source reconstruction in two separate 250 ms time-windows in order to separate brain activity before and after word onset. Significance was assessed employing a non-parametric permutation test. We thus assessed brain activation in comparison with pre-picture baseline power, with permutation statistics^26^ (both phoneme conditions pooled together). In the pre-word interval we found extensive regions of significant amplitude change, spanning bilateral perisylvian areas and frontal and temporal ventral regions (see Fig. 3A top). Within these, a large number of maxima were found (15), so only the four largest were selected for further analysis: left parietal (inferior parietal gyrus: [−50 – 19 27]), right temporal (superior temporal gyrus: [50 0 1]), left fronto-ventral ([−10 29 − 19]), and right temporo-ventral (43 − 31 − 33) (see Table 2 for MNI coordinates). Given their location, it is, however, unclear whether these two last maxima reflect genuine brain activity or source reconstruction artifacts. We then examined the phoneme expectation effect at these sources separating subjects according to experiment, given that moderate evidence for an interaction with temporal predictability was found in the sensor level data (see Fig. 3, panel B top). This revealed that the *fricative* < *plosive* pattern over left-lateralized sources was present in experiment 1 (being maximal over the left parietal and fronto-ventral sources), but minimal or absent in experiment 2. This is consistent with the topographies observed at the sensor level, where differences between ERFs to both phoneme conditions in experiment 1 peaked over mid-left and left anterior areas (see Fig. 2B).

**Table 2:** MNI coordinates of significant maxima for post-word ERF and pre-word TFR sources maps.

*Analysis*	Left hemisphere				Right hemisphere			
	*Location*	*Fig/label*	*MNI coords* [x y z]	*Distance to ERF*	*Location*	*Fig/label*	*MNI coords* [x y z]	*Distance to ERF*
*Post word*								
*ERF*	Parietal	3. b1	[−49−27−26]	—	Temporal	3. b2	(−62−24−09]	—
*Pre word*								
theta	Parietal	5. Θ1	[−43−33−40]	16.4	Parietal	5. Θ3	[−55−30−45]	14.9
	**Temporal**	**5**. **Θ2**	**[−55**−**31**−**15]**	**13**.**2**	**Frontal**	**5**. **Θ4**	**[−59**−**17**−**16]**	**10**.**3**
					Temporal	5. Θ5	[−58−21 0−5]	37.2
alpha	Parietal	5. α1	[−47−30−50]	24.3	Occipital	5. α3	[−31−77−18]	67.1
	Occipital	5. α2	[−14−82 0−6]	72.6	Parietal	5. α4	[−52−30−50]	42.6
beta	Frontal	5. β1	[−34−13−66]	45.0	Occipital	5. β3	[−27−91−08]	75.6
	Occipital	5. β2	[−14−91–01]	77.1				

The last column provides the distance in mm of each TFR location to the peak ERF to the actual word in the same hemisphere. The TFR sources closest to the post-word ERF ones are highlighted in bold.

In the post-word interval, areas of significant amplitude change with respect to baseline spanned a more constrained bilateral peri-sylvian region. Two cortical maxima were found within these regions, in a left-parietal (very close to the one identified previously), and a right superior temporal area (more posterior than the one identified in the pre-word interval (see Table 2). Both these sources showed a *fricative* < *plosive* pattern, in both experiments.

The ERF analysis thus suggests that indeed, the phonological contrast employed elicited significant differences upon word presentation, and that the brain responses to word onset were located in superior temporal gyrus in the right hemisphere, but inferior parietal in the left. Note that this left source was located remotely from early auditory areas, probably because it received a contribution from both auditory information and pre-existing prediction-related activity recruiting more parietal regions. Separate evaluation for the activations in the two experiments (Supplementary Figure 1), revealed two local maxima for experiment 1 in the left hemisphere (*a6*, more parietal, and *a7*, more temporal) and only one peak (*b5*, pointing to temporal regions) in the same hemisphere for experiment 2. It is reasonable to conclude that the left superior temporal regions are involved in both experiments, but the contribution of the parietal source of experiment 1 affected the location of peak response for the two experiments together (illustrated in Fig. 3).

Importantly, the time-course of the evoked activity clearly showed a peak response for plosive condition and a smoother response for the fricative condition at ~100 ms latency that was located in bilateral auditory cortices (data reported in Supplementary Figure 1). This confirms the validity of the phonological contrast (i.e., plosive vs. fricative) used in this study.

### Preparation for expected target words: image-locked time-frequency response

As in the ERF analysis, we assessed the phoneme-expectation effect at the sensor level using all subjects pooled together. Time-frequency responses (TFR) were estimated in a pre-target word interval (from image offset to the minimum interval length: 250 ms to 1500 ms) and at frequencies in the range 3–30 Hz. We then evaluated the presence of an interaction with temporal predictability over the same time-frequency window. Finally, we performed source reconstruction using a minimum variance beamformer to identify the brain regions underpinning the effects identified in sensor data. Based on the literature and on results obtained with ERFs, we expected these to include superior temporal and parietal areas.

**Figure 4:**
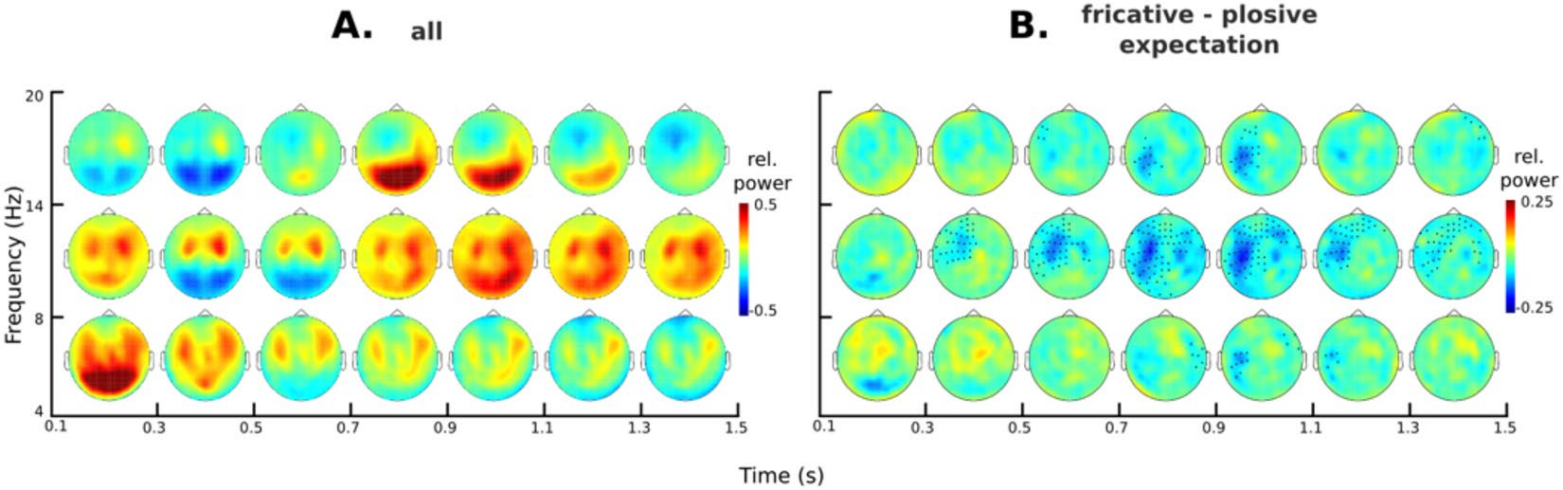
Image-locked time-frequency response. (**A**) Power change with respect to baseline during the analyzed pre-word interval (both experiments pooled together, N = 41). (**B**) Phoneme effect (fricatives minus plosives) during the same interval (N = 41). Sensors that formed part of the cluster with *p* < 0.05 are marked in black.

Figure 4A shows the evolution of relative power with respect to pre-picture baseline as function of time at theta, alpha and beta frequencies. Image offset induced a posterior power decrease at alpha and beta frequencies that turned to power increase at ~700 ms. It also induced a bilateral anterior power increase at theta and alpha frequencies that was sustained throughout the analysis interval at alpha frequencies, but gradually diminished at theta frequencies. There was also a power decrease at beta frequencies over left anterior sensors that intensified as a function of time.

Figure 4B shows the evolution of power differences between fricative and plosive conditions. Cluster based permutations revealed that these conditions differed significantly (largest cluster, *p* = 0.02; second largest cluster, *p* = 1). Differences peaked ~1 s after image onset and spanned mid-left and right middle and anterior sensors, with fricatives displaying a smaller power increase than plosives. The largest identified cluster started 340 ms after image onset, was sustained throughout the analysis interval, and encompassed frequencies from 6 to 20 Hz with a mean of 11 Hz.

Cluster-based permutations comparing the phoneme expectation effect (fricatives minus plosives) between experiments revealed no evidence for an interaction (largest cluster, *p* = 1). In Supplementary Figure 4 we also report the contrast between predictive and non-predictive trials to evaluate the effect of predictability. Strong predictability effects can be observed during the pre-stimulus time interval in all the frequency band described above.

**Figure 5:**
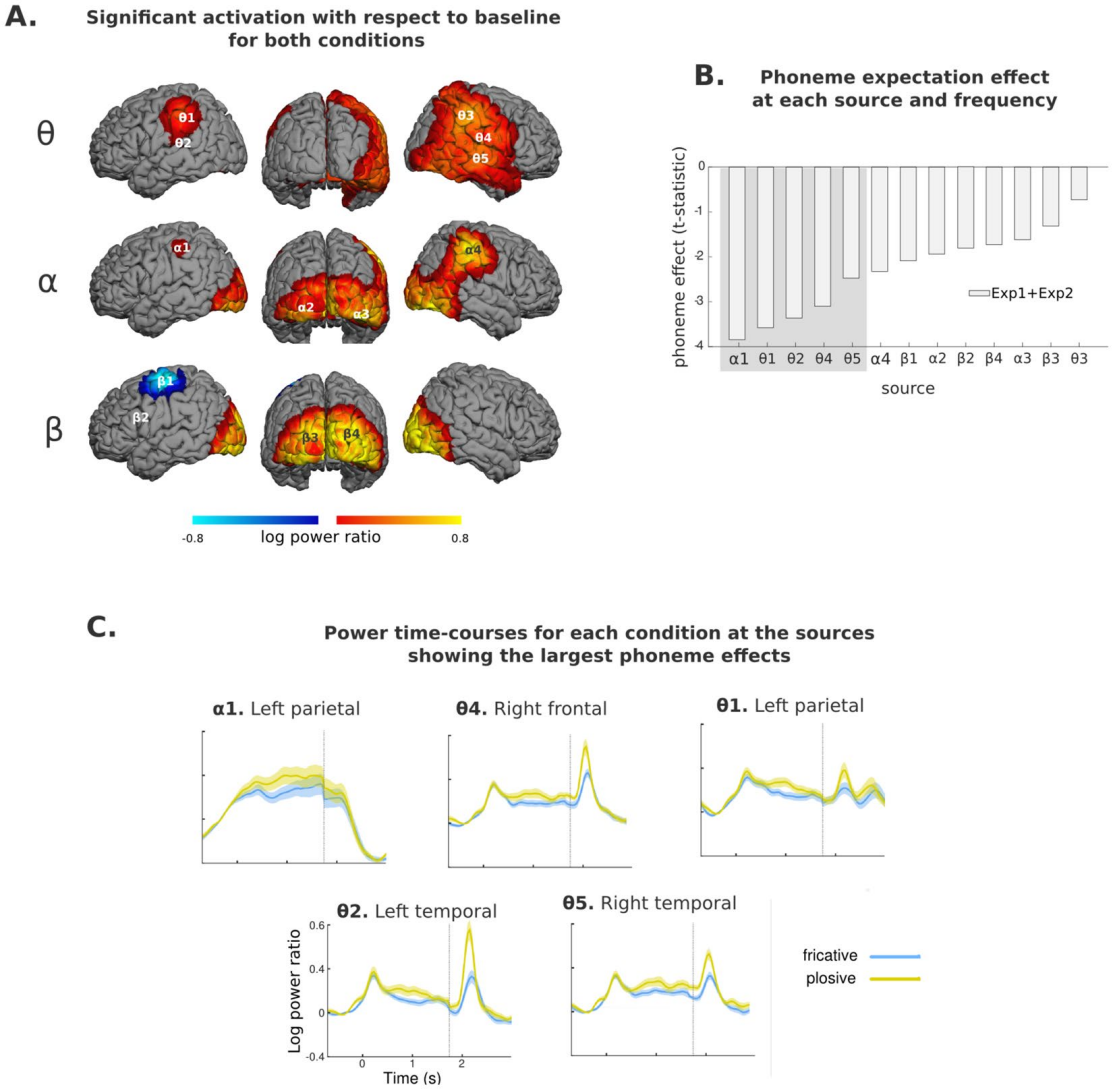
Source reconstruction of TFR effects (both experiments pooled together, N = 41). (**A**) Regions of significant power change with respect to baseline (both conditions) for theta, alpha and beta bands, at 900–1100 ms. The peak activity locations within these areas are marked (Θ1 to β3) within these images. (**B**) Ranking of the phoneme-expectation contrast at each peak according to *t-statistic*, for power averaged across 900–1100 ms. (**C**) Time-course of the whole trial for each phonological expectation condition, for the five sources showing the largest phoneme expectation effect. For visualization purposes, the standard error of the mean for each time-point is depicted as a shaded area around the main line. Vertical dashed lines within these plots indicate a discontinuity in the data shown: to the left, the data reflects the mean calculated using both experiments, to the right, only the data for experiment 2 remains, given that word onset latencies in experiment 1 were variable.

Based on sensor-level results, we evaluated source power at 900–1100 ms, at theta (4–8 Hz), alpha (8–14 Hz), and low-beta (14–20 Hz) frequencies using the data pooled across conditions and experiments. Following the statistical procedures employed for evaluating source level evoked activity, significance was assessed in comparison with baseline power, with permutation statistics. Figure 5A presents the three ensuing power maps, and Table 2 presents the coordinates of local maxima.

The map at alpha frequencies had four clear peaks in inferior parietal and inferior occipital cortex, both bilaterally. In the beta band, there was a frontal peak of power suppression and bilateral occipital peaks of power increase. In the theta band, we found bilateral parietal sources of power increase and a broadly distributed pattern of activity over a perisylvian area in the right hemisphere. Amongst these, we selected the two largest for further analysis (see Fig. 5 panel A), located in temporal and frontal areas (superior temporal sulcus and rolandic operculum respectively).

Although a statistical evaluation of the main effect of temporal predictability was outside the scope of this paper, visual inspection of the sensor level data suggested there may be differences in the topographical distribution of power modulation between experiments (see Supplementary Figure 3). Therefore, as a final check we repeated the analysis procedure described above for each experiment separately. Power spatial distribution in both experiments was very similar (see Supplementary Figure 4) except for a theta source of power increase in left superior temporal cortex that was present in experiment 1, but clearly absent in experiment 2 (with a slight power decrease in this case), and a left inferior frontal beta source of power decrease that was present in experiment 2, but just below threshold in experiment 1. Given the theoretical relevance of both locations, we decided to incorporate the coordinates of these peaks (based on experiment 1 participants in the first case, and experiment 2 participants in the second) to subsequent analysis (with participants from both experiments together).

Considering all frequency bands together, thirteen sources were identified. In order to compare the contribution of each source to the phoneme expectation effect found at the sensor level, we extracted the time-courses at each location, and compared the power difference between conditions averaged over a time interval corresponding to the main cluster of phoneme expectation effect identified in the sensor analysis (0.34 to 1.5 s). Figure 5B shows the standardized difference for each source, sorted according to t-score. Power was higher in the plosive than in the fricative condition in all examined sources (except for one). The largest contributors to the phoneme expectation effect were the left-parietal alpha and right-frontal theta sources. This is largely consistent with the data at the sensor level, where differences between phonemes were concentrated over mid-left areas in the alpha band, but also included right anterior sensors in the alpha and theta bands. Interestingly, the three subsequently-ranked peaks (all in the theta band) were located close to the sources of the ERF to the actual word: the left-parietal and left-temporal sources were 15 mm away from the left-parietal post-word ERF source, and the right temporal sources was less than 15 mm away from the superior temporal post-word ERF source (see Table 2). We thus selected the first five sources for further analysis.

Figure 5C presents the power time-course for each phoneme expectation condition at these five location-frequency pairs for the whole trial duration. Congruently with the sensor-level data, differences between conditions were maximal around 1 s post-image onset and appeared to be sustained until word onset in the right theta sources. Upon word presentation, all sources except the left parietal alpha one displayed a marked power increase and an enhancement of the phoneme effect. This, in conjunction with the spatial proximity of the post-stimulus evoked effect, provides further evidence of their involvement in sensory processing of the word.

## Discussion

In the present study, we aimed to find evidence for predictive pre-activation of auditory words and to explore the oscillatory mechanisms generating them. We compared pre-stimulus activity between two high expectation conditions, that differed only in the phonological features of the expected word (fricative- vs plosive- initial words). In that setting, we found significant differences between expect-fricative and expect-plosive conditions before phoneme onset. Sources of this effect were (amongst others) in superior temporal cortices, associated with phonological processing^20^. Furthermore, these locations were spatially very close to those showing the largest initial event-related response (~100 ms) to the actual word (see Table 2). These results suggest that, at least under high-expectation conditions, prior context may induce the pre-activation of phonologically-detailed word-form representations.

Secondly, we aimed to characterize the oscillatory mechanisms and anatomical networks involved in the generation of prediction process itself. The identified sources encompassed the theta band (although not exclusively) and followed locations along the dorsal speech processing stream^20^, where gradual abstraction from acoustic properties would take place posteriorly from primary auditory cortex across the superior temporal lobe to parietal areas. Therefore, although these sources are not limited to the temporal areas we had hypothesized, they are largely consistent with the pre-activation of the expected phonological features at different levels of representation. These findings extend previous research on lexical prediction claiming that the pre-activation would mainly operate on abstract lexical/semantic information^27^. We here show that, when expectations are precise, pre-activation can percolate down to early sensory brain regions thus possibly pre-activating phonological form-related representations^13,28^.

Interestingly, the theta sources are distributed mainly over the right hemisphere. This is consistent with studies looking at cortical entrainment to speech and non-speech auditory streams, that show stronger theta coherence over right- than left- superior temporal cortices^29^. The involvement of the theta band in the pre-activation process is also in agreement with studies looking at the pre-target word interval in the visual domain^7,13^. While in these previous studies theta band activity could also be linked to lexical retrieval (given the predictive vs non- (or less-) predictive contrasts), in our case it can be more directly linked to a pre-activation at the sensory (phonological) level.

This prominence of theta oscillations in our results may appear to be at odds with the large body of evidence linking beta oscillations to predictive processes in general^24,30,31^ and specifically within the speech processing literature^17,18^. But in fact, our results did reveal significant beta desynchronization with respect to baseline over left frontal regions, that did not differ between expected phonemes. This is actually not unexpected: Cope and colleagues directly link beta oscillations to the precision of the generated predictions, and our main experimental conditions do not differ in this respect. Our data thus suggests that even if the generation of predictions might involve left-lateralized frontal beta desynchronization, it has bilateral effects upon sensory cortices involving theta band oscillations.

Within the theta band sources, the right frontal motor source displayed the largest phoneme expectation effect. Its location, in the Rolandic operculum, is related to tongue motor control and speech articulation^32^. Although controversial, the role of motor areas in passive speech perception is a recurrent topic in speech processing research^33,34^. Interestingly, the involvement of the motor system in perception could be mediated by the prediction process itself, that according to some accounts^35–37^ would use motor simulations to generate representations of their sensory effects^38,39^, or possibly of their temporal dynamics^40^.

The strongest phoneme expectation effect occurred in a left inferior parietal alpha source but its time-course (Fig. 5C) suggests that it does not reflect phonological pre-activation. If, in our results, differences in the inferior parietal area were reflecting a phonological effect (as suggested in^41^), we would have expected an enhancement of such a difference, and of overall activity with respect to baseline, once the actual phonological information was available, at word onset. Whilst this was true of the theta sources, it was not the case in the alpha left parietal location (compare time-course *α1* with *θ2* and *θ5* in Fig. 5).

The left parietal alpha source could, rather, subserve temporal prediction^42^. This area would estimate the timing of the expected target occurrence, to be used as a top-down attentional filter to optimize processing of expected targets. This idea of the attentional filter is congruent with an effect in the alpha band, given its role in attentional selection^22,43^. Based on the pre-activation of the abstract phonemic representation we propose, the system could recruit different attentional resources for the two categories (fricatives are relatively independent of temporal cues, while plosives consist of several “acoustic phases” whose temporal durations are relevant for determining their specific identity). In this way, activity in the left inferior parietal cortex could be functionally segregated in distinct frequency bands: a time-based attentional filter, implemented through alpha band activity, and a linguistic pre-activation of the expected target implemented in the theta band. Although both cognitive processes may exert top-down influences on perception, they fulfill separable functions^44^.

It is important to acknowledge two main limitations of the present study. Firstly, all our effects could be attributed to differences in timing, rather than phoneme identity. Indeed, participants’ task was to detect spelling errors in the vowel following the first phoneme. However, this second phoneme arrived systematically later, and with more variable onset, in the fricative than in the plosive condition. Hence, in addition to the obvious difference in phoneme identity, our conditions also differed in the time-predictability of the onset of the task-relevant vowel. It is important to note however that even in this case, the different task requirements are mainly driven by the fact that the system “realizes” the identity of the (yet-to-come) first phoneme before its appearance. Secondly, the source contributing most to the significant phoneme expectation effect observed at the sensors was the alpha parietal location, in our interpretation responsible for implementing an attentional filter, rather than a predictive pre-activation. The theta effects observed at the source level alone might have been insufficient to generate a statistically significant effect at the sensors. The subtlety of these effects could be in part responsible for the scarcity of pre-target word evidence in the literature and should be taken into account in future studies.

In the present study, we also assessed to what extent temporal uncertainty regarding word onset modulated phoneme pre-activation mechanisms. Although we found no evidence for the influence of word timing on the difference between phoneme expectation conditions, the evoked response during the peri-word interval suggests that such an interaction was indeed present. Therefore, even if pre-activation occurs in both cases, the nature of the representations, or the processes generating them may be modulated by the predictability of its temporal onset. We suggest that the differences in the phoneme expectation effect found in each experiment may be due to attentional selection processes^45^ that would be present in experiment 1, on top of predictive pre-activation that would be present in both experiments. This attentional selection would be implemented through evoked activity in parietal areas, in the form of a sustained differential activity as a function of expected phoneme. Somewhat intriguingly, this sustained attentional selection would not be implemented when both the identity and the timing of the target word could be predicted, with phoneme related differential activity occurring only “just in time” in this case. These findings converge with Cope *et al*. evidence of predictions instantiated right before the onset of the expected auditory stimulus.

In sum, our results show that the word-form features of expected phonemes modulate anticipatory activity over several different anatomical locations and frequency bands. This is in line with the view that predictive mechanisms involve brain-wide networks. Crucially, temporal auditory areas were an important part of this network, showing that phonological representations were activated before word onset.

## Methods

### Participants

Twenty-one right-handed native Spanish speakers took part in the first experiment and twenty-six in the second (aged between 19 and 40, mean 24; and 20 to 39, mean 25 respectively). From the first experiment one participant was excluded due to excessive noise in the recordings, and from the second, one was excluded due to lack of compliance with experimental instructions, two due to excessive noise and artifacts, and two due to technical problems with the audio system detected after the experiment. This left 20 participants in the first experiment, and 21 in the second. Their vision was normal or corrected to normal and they had no history of neurological disease. All participants provided informed consent in accord with the Declaration of Helsinki before starting the experiment and received €10 in exchange for their collaboration. The present study was approved by the BCBL Ethics Committee.

### Stimuli and procedure

A set of 20 pictures depicting fricative- and stop-consonant-initial words in Spanish were selected from the Bank of Standardized Stimuli (BOSS^46^). The color pictures obtained were transformed to grayscale, trimmed, resized to a target diagonal of 500 pixels, and placed on a 550 by 550 pixel square with a medium-gray background using the imageMagick software package. The pictures were balanced across conditions with respect to several ratings from the BOSS database (name-, object-, and viewpoint-agreement, familiarity, subjective complexity and manipulability), as well as several lower level image properties (contour complexity, number of pixels, and brightness). These properties were evaluated with Matlab 2012b and imageMagick software packages. The corresponding words were 5–6 letters long and were balanced across conditions on frequency (obtained from the esPal database^47^), length in syllables, and semantic category (natural vs artifact). All words started with a consonant followed by a vowel (see Table 3 for full stimuli list). Words were also balanced in a set of phonological dimensions: number of phonemes (plosives: 5.6, SD: 0.52; fricatives: 5.4, SD: 0.90; p = 0.62), number of syllables (plosives: 2.7, SD: 0.48; fricatives: 2.5, SD: 0.52; p = 0.45), position of the accented syllable (plosives: 1.9, SD: 0.32; fricatives: 1.7, SD: 0.66; p = 0.45), phonological neighbors (none for both groups). An additional non-predictive condition was included in the experimental materials, by using scrambled pictures as cues.

**Table 3:** Items in each phoneme condition. English translations provided within parenthesis.

Plosive-initial	Fricative-initial
toldo (awning)	sillón (armchair)
pelota (ball)	silla (chair)
camión (truck)	cereza (cherry)
cañón (cannon)	secador (hairdryer)
conejo (rabbit)	farola (streetlamp)
cometa (kite)	zapato (shoe)
camisa (shirt)	zorro (fox)
cohete (rocket)	salero (saltshaker)
tomate (tomato)	sierra (saw)
toalla (towel)	zueco (wooden shoe)

In total, 360 response trials were generated (120 in each condition: plosive-predictive, fricative-predictive, non-predictive). Trial order was pseudo-randomized, avoiding more than three repetitions in a row of the same image cue.

The picture was followed by an auditory word that was correctly pronounced in 50% of the trials, and incorrectly otherwise. In the latter case, the vowel following the first consonant was substituted by another vowel, always creating a pseudo-word. Three different mispronunciations were generated for each actual word when possible (for some words only one pseudo-word could be generated).

The auditory stimuli were created by recording a female native Spanish speaker reading aloud the target words (both the correct and mispronounced versions) in a sound-proof cabin. For each item multiple versions of the word were recorded following a random order, and one exemplar of each was chosen manually. The recordings were cut and equalized to 70 dB using Praat^48^.

Participants were instructed to evaluate whether the word was correctly pronounced or not and were informed that the incorrectly pronounced words had one phoneme replaced. They were encouraged to pay attention to the preceding images, explaining that these would always give valid cues as to the upcoming words. Participants responded with a left/right index button press, with yes/no response side counterbalanced across participants. Each trial started with a blank screen presented for a variable interval from 700 to 1200 ms, followed by the image-cue for 250 ms, and after a fixed (1750 ms: experiment 2) or variable (1250 to 2250 s: experiment 1) interval, the word was presented auditorily. Participants had a maximum of 500 ms to give a response, and visual feedback was provided after incorrect trials (red cross in the center of the screen for 100 ms).

In addition to the response trials, there were 40 catch trials. These were identical to the experimental trials, but the image presented was the original color-version and participants were instructed not to respond to the word in these cases. These were included to make sure that participants were attending the image-cue, and not just waiting for the word.

Auditory stimuli were presented through plastic tubes and silicon earpieces to participants’ ears. Visual stimuli were presented within a 550 × 550 px medium-gray square against a black background, on a back-projection screen situated 150 cm away from the participant. The experimental block lasted approximately 30 minutes. Participant-controlled pauses were provided every 10 trials, in addition to two experimenter-controlled ones. The experimental session included another 30 minutes block with a similar paradigm in which target words were presented in written form rather than auditorily. However, in the present paper we report the results for the auditory block only. Order of presentation of these two blocks was counterbalanced across participants. Between them, 10 minutes of resting state were recorded. Overall, the recording session lasted approximately two hours.

### MEG data acquisition

Brain activity was recorded in a magnetically shielded room using a whole head MEG system (Vectorview, Elekta/Neuromag) with 306 sensors arranged in triplets comprised of one magnetometer and two orthogonal planar gradiometers. Participants were screened for magnetic interference prior to data collection and instructed to limit head and face movements as much as possible, and to fixate on the center of the screen. Data was acquired with a 1000 Hz sampling rate and filtered during recording with a high-pass cutoff at 0.1 Hz and a low-pass cutoff at 330 Hz via the Elekta acquisition software. Head movements were monitored continuously using five head position indicator coils attached to the participant’s head. Their location relative to fiducials (nasion and left and right pre-auricular points) was recorded at the beginning of the session using an Isotrak 3-D digitizer (Fastrak Polhemus, USA). In addition, head shape was digitized to allow for alignment to each subject’s structural MRI for subsequent source localization. Eye movements and heartbeats were monitored using vertical and horizontal bipolar electro-oculograms (EOG) and electrocardiogram (ECG).

### MRI data acquisition

Participants’ high-resolution 3D structural MRIs (T1-weighted MPRAGE sequence) were acquired with a 3 T Trio scanner (Siemens, Munich, Germany) and a 32-channel head coil. To limit head movement, the area between participants’ heads and the head coil was padded with foam and participants were asked to remain as still as possible. Snugly fitting headphones were used to dampen background scanner noise and to enable communication with experimenters while in the scanner.

### MEG data preprocessing

MEG data were initially preprocessed using Elekta’s MaxFilter 2.2 software, including head movement compensation, down-sampling to 250 Hz, and noise reduction using signal space separation method^49^ and its temporal extension (tSSS) for removing nearby artifacts^50^. Manually-tagged bad channels were substituted by interpolated values.

Subsequent data analysis was carried out in Matlab 2012b, using the FieldTrip toolbox^51^. The recordings were segmented from −1000 ms to 4000 ms (experiment 1) or 3500 ms (experiment 2) relative to the presentation of the picture, and low-pass filtered at 100 Hz. Since in experiment 1 the delay between the cue and the target was variable, trials were then trimmed to exclude the response to the actual word. This allowed the generation of an image-locked average containing only preparatory activity. In addition, trials for this experiment were re-segmented time-locked to word presentation, in order to allow examination of the event-related response to the actual word.

Eye movement, blink and electrocardiographic artifacts were linearly subtracted from recordings using independent component analysis (ICA)^52^. ICA components responsible for eye movements were identified calculating correlation values between the component signal and the activity of the VEOG/HEOG and the ECG channels with subsequent visual inspection to remove any epochs with remaining artifacts. Fifteen percent of the trials were rejected in experiment 1, and 9% in experiment 2. There were significant differences in trial rejection between experiments (*F*(1,39) = 11.0, *p* = 0.002) but not between phoneme expectation conditions (*F*(1,39) = 1.2, *p* = 0.3). Also, phoneme expectation conditions and experiments did not interact for the number of rejected trials (*F*(1,39) = 0.9, *p* = 0.3). Further sensor-data analysis was performed using gradiometer data only, but both magnetometer and gradiometer data were used for source localization.

### Experimental design

Before conducting analysis of the neural response we examined the effect of our experimental manipulations on reaction times to the actual words to establish the presence of predictability and mismatch effects using mixed models with crossed random effects for items and subjects^53^.

We conducted analysis of the MEG data in two steps: firstly, statistical inference was carried out on sensor level data to establish the presence of the effects of interest. Secondly, identified effects were localized in the brain using a whole-brain source reconstruction approach.

Statistical inference was carried out using cluster-based permutation tests. Two different comparisons were performed. Firstly, the presence of a phoneme expectation effect (fricatives vs plosives) was assessed using the 41 subjects of both experiments pooled together. Secondly, the presence of an interaction between phoneme expectation and temporal predictability was evaluated by contrasting the difference between fricatives and plosives for subjects in experiment 1 (variable interval, *N* = 20) to those in experiment 2 (fixed interval, *N* = 21).

### Behavioral analysis

Behavioral data were analyzed using the free software statistical package R^54^, and the lme4^55^ library. Given the controversy behind calculating degrees of freedom and corresponding p-values in these types of models, we evaluated the significance of predictors using the normal approximation (|t > 2|).

Trials with incorrect responses and reaction times (RT) under 0.2 s were removed before model fitting. The resulting RTs served as the dependent variable against mixed effects multiple regression models were built. Our independent variables of interest included the following bivariate categorical variables: Phoneme (fricative or plosive), Image cue (predictive or nonpredictive), Pronunciation (correct or incorrect) and Experiment (1 or 2). These variables were coded using treatment coding, making plosive, predictive, correct, Experiment 1 trials as the reference level.

### Sensor level analysis

#### Event related fields

The word-locked epochs for each experiment were low pass filtered at 35 Hz. The uncombined gradiometer signals were then averaged according to experimental condition, and baseline corrected using a 500 ms window prior to image onset (in order to preserve phoneme-expectation differences that were hypothesized to be present before word onset). Finally, the orthogonal directions of each gradiometer pair were combined using the Euclidean norm.

#### Time-frequency

Time-frequency representations over 3–30 Hz were obtained for the image-locked epochs in each experiment using a frequency-independent Hanning taper and a 500 ms sliding window advancing in 40-ms steps, giving rise to a 2-Hz frequency resolution. Power estimates were then separated into the two phonological expectation conditions and averaged over trials. For each gradiometer pair, power was averaged across the two sensors, resulting in 102 time-frequency power maps. Power was then normalized by its baseline value (450 to 250 ms prior to picture presentation).

#### Statistical analysis

Differences between conditions were assessed using cluster-based permutation tests^56^. This analysis controls for multiple comparisons whilst maintaining sensitivity by taking into account the temporal, spatial and, for time-frequency data only, frequency dependency of neighbouring samples. First, the data were clustered by performing pairwise comparisons (t-tests) between each sample (time-frequency-sensor or time-sensor point) in two conditions. Contiguous values exceeding a *p* = 0.05 threshold were grouped in clusters, and a cluster-based statistic was derived by adding the t-values within each cluster. Then, a null distribution assuming full exchangeability (i.e. no difference between conditions) was approximated by drawing 3000 random permutations of the observed data and calculating the cluster-level statistics for each randomization. Finally, the cluster-level statistics observed in the actual data were evaluated under this null distribution.

Dependent-samples t-tests were employed for the sample-pairwise comparisons in the phoneme effect analysis (fricatives vs plosives, within-subjects contrast over 41 subjects), whereas independent-samples t-tests were used in the analysis for the interaction between phoneme and temporal uncertainty (experiment 1 vs experiment 2, between-subjects contrast, 20 and 21 subjects respectively).

For the event-related fields, a 500 ms second window centered around word onset was statistically analyzed, in order to include early responses to the word, and activity just prior to it. For the time-frequency data, the statistical analysis was performed in a time window ranging from image offset (250 ms) to the minimum trial length in experiment 1 (1500 ms).

### Source reconstruction

Source reconstruction was carried out to identify the brain areas underpinning the experimental effects detected at the sensor level, both for the event-related fields and the time-frequency data.

Different source reconstruction approaches were implemented for each kind of data: a minimum-norm estimate (MNE^57^) for the event-related fields, and a linearly constrained minimum variance beamformer (LCMVB^58^) for the time-frequency data. Although theoretically there are no reasons to prefer different models for each type of data, from a practical perspective beamforming may work better for sustained brain responses (such as induced modulations of ongoing oscillations) and MNE for shorter-lived responses as evoked by a stimulus^59^.

Participants’ high-resolution 3D structural MRIs (T1-weighted) were segmented using Freesurfer software^57,60,61^. The MRI and MEG coordinate systems were co-registered using the three anatomical fiducial points for initial estimation and the head-surface points for manual adjustment of the surface co-registration. We then computed realistic head-models (one-shell boundary element) using these segmented T1 images. The MRI was missing for one participant and we therefore used a template head model in this case. The forward model was computed for three orthogonal source orientations, placed on a 5 mm grid covering the whole brain using MNE suite^62^ (Martinos Centre for Biomedical Imaging). Each source was then reduced to its two principal components of highest singular value, which closely correspond to sources tangential to the skull. Both planar gradiometers and magnetometers were used for inverse modeling after dividing each sensor signal (and the corresponding forward-model coefficients) by its noise standard deviation. The noise variance was estimated from the 500-ms baseline prior to picture onsets in all conditions.

#### Event related fields

Windows for source localization were selected a-priori, to reflect activity just before and just after word presentation (250 ms each). An MNE inverse solution^57^ was used to project the ERF data into source space, using a noise co-variance matrix estimated from a 500 ms pre-image baseline. Source power in the two windows of interest (250 ms before and 250 after word presentation) was then averaged and normalized by the power in the baseline. In order to allow for subsequent group level analysis, the individual MRIs were mapped to the standard Montreal Neurological Institute (MNI) brain through a non-linear transformation using the spatial-normalization algorithm implemented in Statistical Parametric Mapping (SPM8^63,64^), and the ensuing spatial transformations were applied to individual maps.

We located regions of peak activity with respect to baseline and restricted between-condition comparisons to those sites (see^65^). Non-parametric permutation tests^26^ were used to identify regions of significant change with respect to baseline, using both predictive conditions. Within these regions, we identified the coordinates of local maxima (sets of contiguous voxels displaying higher power than all other neighboring voxels). Any maxima located in deep brain structures were discarded due to their probable artifactual nature. In practice, subject- and group-level baseline power maps were computed as done for the maps reflecting trial-dependent activity. Group-level difference maps were obtained by subtracting f-transformed trial and baseline group-level power maps. Under the null hypothesis that power maps are the same whatever the experimental condition, the labeling trial and baseline are exchangeable at the subject-level prior to group-level difference map computation. To reject this hypothesis and to compute a threshold of statistical significance for the correctly labeled difference map, the permutation distribution of the maximum of the difference map’s absolute value was computed for all possible 4096 (=212) permutations. The threshold at p < 0.05 was computed as the 95-percentile of the permutation distribution^26^. All supra-threshold local coherence maxima were interpreted as indicative of brain regions showing statistically significant activity elicited by our experimental manipulation. In order to estimate which of the identified cortical sources contributed more to the phoneme effects detected at sensor level, we then calculated the t-statistic for the phoneme condition contrast (fricatives *vs*. plosives) at each source and ranked the sources accordingly.

#### Time-frequency

Power and cross-spectral density (CSD) matrices were estimated in the time-frequency windows selected on the basis of the sensor-level statistical analysis, and in an equally-sized baseline period prior to image onset. The real part of the combined CSD matrices from both the baseline and the time window of interest in all conditions was used to compute an LCMVB^58^ beamformer.

Subsequent group-level analysis proceeded as with the ERF data. In this case, permutation tests were performed on log-transformed power ratios (activity of interest to baseline). Peak coordinates of power-ratio were then identified. The relative contribution of each source to the phoneme effect was explored by examining the standardized mean difference between fricative and plosive expectation conditions at each peak source.

Finally, in order to explore the temporal evolution of power at the identified sources over the whole trial, we extracted the time courses for these peaks. We used a forward solution restricted to these locations and CSD matrix covering the frequencies of interest and the whole trial length to project the time domain data to these sources. We then used the same parameters for spectral decomposition employed in the sensor level analysis, to obtain the time-course for each frequency band of interest and each phoneme expectation condition at each peak activation location.

### Data availability

The datasets generated during and/or analysed during the current study are available from the corresponding author on reasonable request.

## Electronic supplementary material

Supplementary Figures
