# Supplementary material for: Theta oscillations mediate pre-activation of highly expected word initial phonemes
Source: Sci Rep. 2018 Jun 22;8:9503. doi: 10.1038/s41598-018-27898-w (PMC6015046; doi:10.1038/s41598-018-27898-w)

Supplementary material

Theta oscillations mediate pre-activation of highly expected word initial phonemes
Irene F. Monsalve, Mathieu Bourguignon & Nicola Molinaro

Supplementary Figure 1: Source reconstruction of ERF effects for each experiment separately. Regions of significant power change with respect to baseline (both conditions) at 250 ms windows before and after word onset.

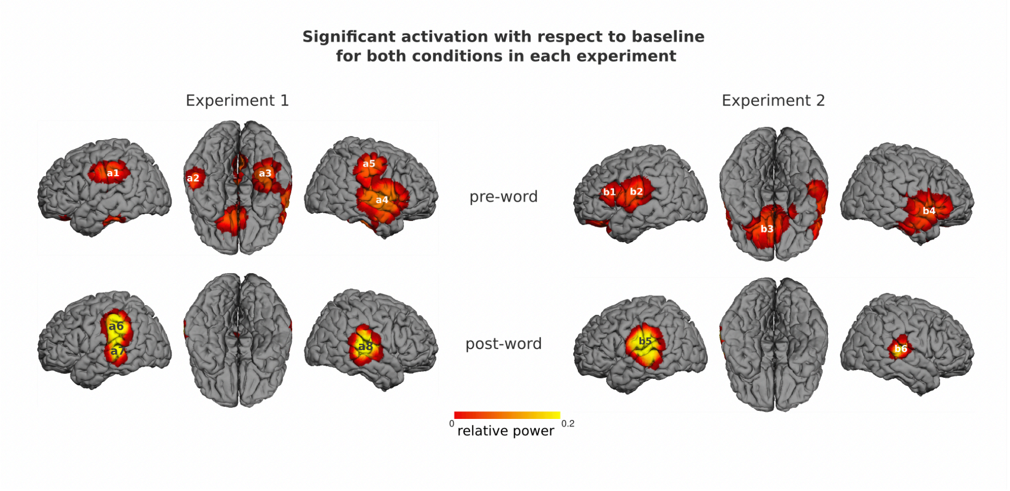

Supplementary Figure 2: Predictive minus non-predictive contrast. Sensors part of the significant clusters detected in the analysis are marked in black. Cluster-based permutations highlighted the statistical reliability of the following clusters: first positive cluster (13-19 Hz, 0.74-1.5 sec): $p=0.0039$; second positive cluster (3-7 Hz, 0.26-1.42 sec): $p=0.045$; negative cluster (5-29 Hz, 0.26-1.38 sec): $p<0.001$.

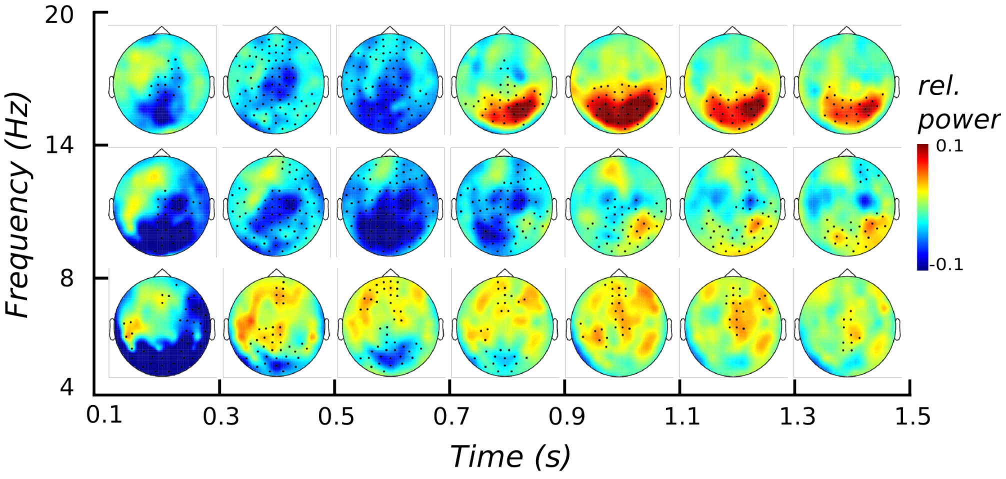

Supplementary Figure 3: Image-locked TFR response for each experiment separately. Relative power change with respect to baseline during the pre-word interval for Experiment 1 (left) and Experiment 2 (right).

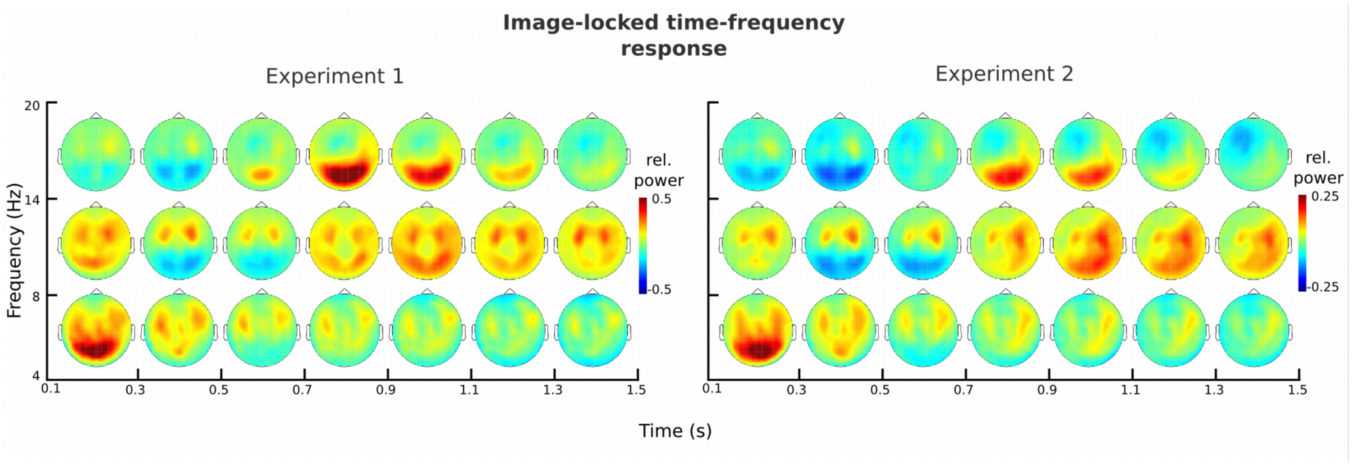

Supplementary Figure 4: Source reconstruction of TFR effects for each experiment separately. Regions of significant power change with respect to baseline (both conditions) for theta, alpha and beta bands, at 900-1100 ms.

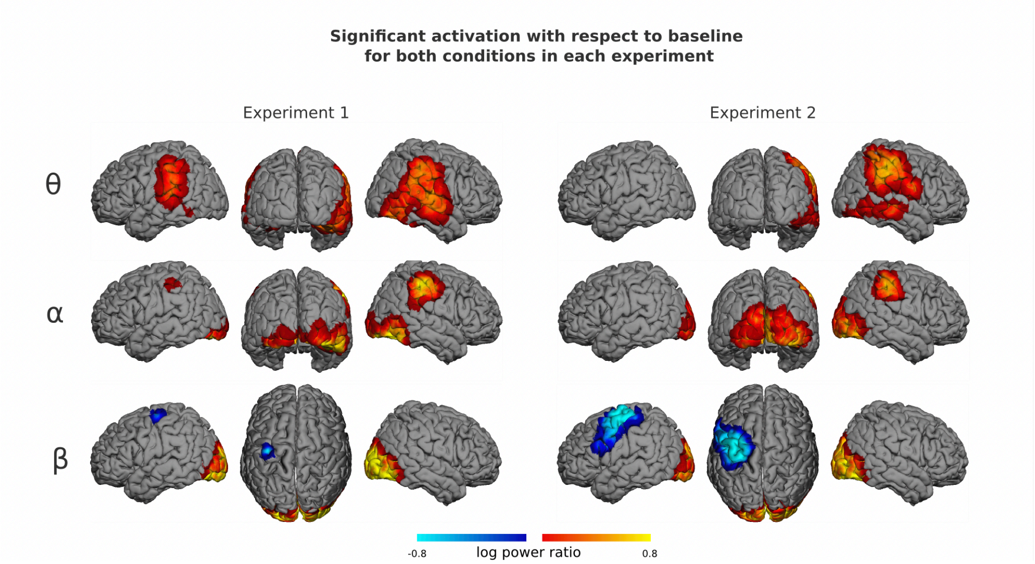

Supplement: Supplementary file 1 — Supplementary Figures [file 41598_2018_27898_MOESM1_ESM.pdf]
